# An Oleaginous Element Indispensable in Phthisis

**Published:** 1855-09

**Authors:** J. A. Damour

**Affiliations:** Macon, Ga.


ARTICLE VIII.—"*An Oleaginous Element Indispensable in Phthisis.*" By J. A. DAMOUR, M. D., Macon, Ga.

MESSRS. EDITORS: I would offer for the consideration of the public a few remarks on the important and sadly neglected subject of Diet.

The health and longevity of man depend in a great measure on the character of the aliment he ingests, and though it would seem that this fact must need little urging to attract the amount of attention it deserves, yet as I think none will deny the practical results which should follow conviction of the judgment on this point, it has not as yet to any extent been observen in any community.

I do not wish to enter on the discussion of the question as to whether an animal or a vegetable diet is the more healthy. I would merely state, in regard to this much vexed question, that at the present time it is the conviction of all sound medical men that a mixed diet is the most conducive to health, mental and physical, at least in the Temperate zone, with which we are most nearly concerned.

My design in writing this brief communication is to urge on the public the startling fact which no one, as far as my knowledge extends, has noticed, but which, nevertheless, will be found to be true, that thousands of human beings die annually in the temperate regions from the sole cause that they will not eat oily nutriment.

An approximation to this truth has been made, it is true, in the belief in the efficacy in "Cod Liver Oil;" but merely an approximation, inasmuch as its therepeutical power is not attributed to its oleaginous character—its union of carbon with the blood—but to the presence of iodine, an alterative, it is true, of value. The medicinal virtues of this article, especially in scrofulous cases, as a preventive or remedy in certain stages of consumption is due, in my humble opinion, solely to the fact of its supplying carbon and nutriment to the blood, which, in such subjects, are invariably found wanting.

From observation, I have come to the conclusion that any food of an oily nature, such as the fat of mutton, beef, &c., will answer all the purposes for which the Cod Liver Oil is used; and this fact, if it shall be accepted, is one of value, as the cost of the vaunted oil of the cod is very great.

It will be found on investigation that those nations who use the most food containing the oleaginous element are the most free from Tubercular Phthisis, and that in this community few if any reach the age of fifty who do not eat fat meat.

The rationale of the process by which oily food is thus efficacious is briefly this: It unites and affords carbon to the system, and has for its function to protect the organs; maintain their temperature; and to serve for nutrition in case of need, as is observed in torpid animals.

Every well-informed physician admits that the fatty matters of the chyle and blood give nutrition to the adipose and nervous tissues, and we also maintain that animal heat is generated by the same. The calorifying process seems essentially to consist in the union of the carbon and hydrogen of fatty and other combustible matters, with oxygen introduced by the respiratory process, thus generating carbonic acid and water, which are set free through the same channel. But there is strong reason to believe that fatty matter performs a most important part in the assimilation of even the albuminous constituents of the food, and in their conversion into plastic material in the first place, and subsequently into actual tissue. It has been shown by Lehmann and Elsasser that the combination of fat with protein compounds renders the latter much more easily reducible by the digestive process; and it has also been shown by Lehmann that the presence of fat is necessary to enable albuminous matters to act as ferments, whence he arrives at the conclusion that "fat is one of the most active agents in the metamorphosis ot animal matter."

As I before asserted, the remarkable power which "Cod Liver Oil" has been found to possess, of promoting the nutrient processes in individuals laboring under tuberculous cachexia, can scarcely be attributed to anything else than to its furnishing an abundant supply of fatty matters in a form that renders them peculiarly easy of assimilation. Taking all these facts into consideration, we seem justified in accepting the conclusion that in the metamorphosis of the albuminous constituents of the food into the organized tissues, of which they are the proper pabulum, fat takes as essential part; and thus it comes to have a much higher place in the economy of the living system than has usually been assigned to it.

